# Rescoring Peptide Spectrum Matches: Boosting Proteomics Performance by Integrating Peptide Property Predictors Into Peptide Identification

**DOI:** 10.1016/j.mcpro.2024.100798

**Published:** 2024-06-11

**Authors:** Mostafa Kalhor, Joel Lapin, Mario Picciani, Mathias Wilhelm

**Affiliations:** 1 Computational Mass Spectrometry, TUM School of Life Sciences, Technical University of Munich, Freising, Germany; 2 Munich Data Science Institute, Technical University of Munich, Garching, Germany

**Keywords:** peptide identification, rescoring, data-driven rescoring, machine learning, artificial intelligence, peptide property prediction, computational proteomics

## Abstract

Rescoring of peptide spectrum matches originating from database search engines enabled by peptide property predictors is exceeding the performance of peptide identification from traditional database search engines. In contrast to the peptide spectrum match scores calculated by traditional database search engines, rescoring peptide spectrum matches generates scores based on comparing observed and predicted peptide properties, such as fragment ion intensities and retention times. These newly generated scores enable a more efficient discrimination between correct and incorrect peptide spectrum matches. This approach was shown to lead to substantial improvements in the number of confidently identified peptides, facilitating the analysis of challenging datasets in various fields such as immunopeptidomics, metaproteomics, proteogenomics, and single-cell proteomics. In this review, we summarize the key elements leading up to the recent introduction of multiple data-driven rescoring pipelines. We provide an overview of relevant post-processing rescoring tools, introduce prominent data-driven rescoring pipelines for various applications, and highlight limitations, opportunities, and future perspectives of this approach and its impact on mass spectrometry-based proteomics.

## Highlights

• Overview of common post-processors and data-driven rescoring pipelines.
• Rescoring integrates advanced peptide property predictions into database searching.
• Data-driven rescoring enhances match discrimination, boosting identification rates.
• Overview of challenges, opportunities, and perspective of data-driven rescoring.

## Graphical Abstract

**Figure:**
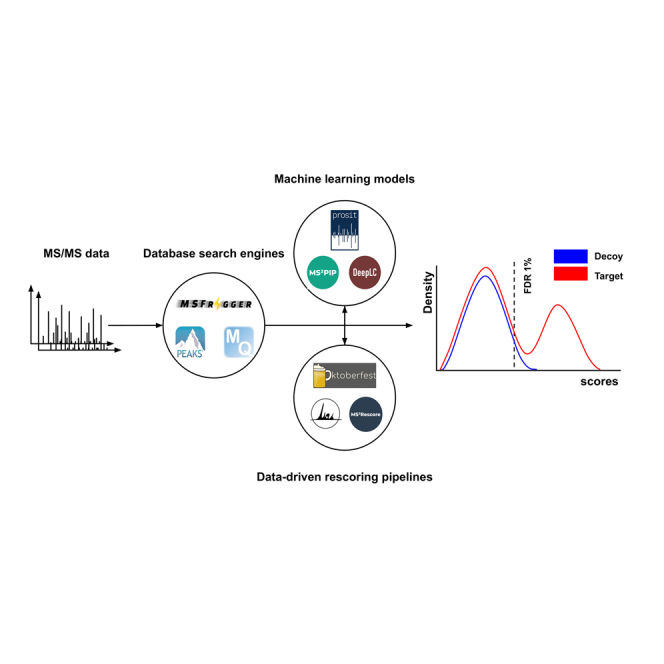

## In brief

Rescoring peptide spectrum matches by integrating predicted peptide properties, such as MS/MS intensity and retention time, substantially boosts the number of confidently identified peptides over traditional database search engines. This approach facilitates data analysis of challenging datasets in various fields, including immunopeptidomics, metaproteomics, and single-cell proteomics. Here, we introduce well-known post-processing rescoring tools and recently developed data-driven rescoring pipelines, followed by discussing their imitations, potential, and future perspectives.

Liquid chromatography-tandem mass spectrometry (LC-MS/MS) systems have recently seen tremendous progress in various aspects of their application, such as sensitivity, and speed. For example, recently developed mass spectrometers such as the Orbitrap Astral (1) and timsTOF Ultra (2) can achieve hundreds of MS/MS scans per second. Such advances further increase the demand for accurate MS data-processing approaches supporting high throughput and high-quality peptide identification, that is, by increasing true positive identifications while possibly reducing false positives. This is particularly crucial when analyzing data in health-related domains (3, 4).

The most common approach for peptide identification in data-dependent acquisition (DDA) remains database searching using so-called database search engines (DBSE) (5, 6). Briefly, DBSEs mimic the bottom-up proteomics workflow by in silico digestion of a user-provided protein sequence database to create peptide candidates matching the expected precursor mass and charge state estimate of a relevant MS/MS scan. For each peptide candidate, a theoretical spectrum is generated, matched, and scored against the experimental spectrum. A variety of different scoring functions have been developed that aim to distinguish between correct and incorrect matches (7, 8) such that the most likely correct peptide is ranked first when multiple peptide candidates are scored against a spectrum. To control the false discovery rate (FDR), known incorrect peptides, so-called decoys, are added to the search space that mimics the distribution of incorrect matches in the target search space, known as the target-decoy approach (TDA) (9). The resulting list of peptide-spectrum matches (PSMs) can then be sorted and filtered for a desired FDR such that the resulting list of identified peptides and proteins contains at most a certain desired percentage of false matches.

Most DBSEs generate multiple scores, with each score aiming to capture a specific aspect of the quality of a PSM. As a result, applying and controlling the FDR cannot be achieved by simple sorting of one score since these scores are typically not well-calibrated, making their direct comparability challenging (10). To benefit and integrate multiple scores or other peptide characteristics such as length, the concept of rescoring was introduced (11, 12). Today, rescoring DBSE results is a commonly applied post-processing (post-processor rescoring; PPR) step in all state-of-the-art workflows and has massively contributed to the increase in the number of confidently identified PSMs, peptides, and proteins over the last decades.

Despite the advances in scoring PSMs, the majority of DBSEs ignore or only partially integrate measurable properties of peptides, such as fragment ion intensity and retention time (RT), limiting their ability to efficiently separate correct from incorrect matches. One option to include fragment ion intensities is spectra library search engines (SLSE), where previously identified MS/MS spectra are collected and used to identify these peptides in new data (13, 14). With the advent of machine learning (ML) in proteomics, various models have been developed that can accurately predict peptide properties, such as fragment ion intensities (15) and their retention time (16). First attempts included decision trees (17) and single-layer neural networks (18). With recent advances in deep learning, more accurate models for fragment ion intensities, such as Prosit (19) and pDeep (20), and other features such as retention time (21) and binding affinity (BA) (22) are now available. As a result of highly accurate peptide property predictions, matching experimental data to predictions can be done at an unprecedented quality, and integrating these predictions into scoring processes revived the development of rescoring pipelines (23, 24, 25, 26). Particularly in the last few years, the integration of predictions, here referred to as data-driven rescoring (DDR), has been shown to substantially increase the number of identifications in otherwise challenging applications of MS, for example, in immunopeptidomics (27, 28), single-cell proteomics (29), and metaproteomics (19).

This review introduces the concepts of rescoring and its future potential. We start by introducing the concept of PPR, provide a history of the most commonly used tools applied in bottom-up data-dependent acquisition-based proteomics analysis pipelines, present the current state-of-the-art DDR pipelines, and close with a perspective on the topic of MS data analysis assisted by machine learning. Detailed descriptions of machine learning models (30, 31) and their use cases in proteomics, such as spectral library generation (32), and the application of rescoring specifically in immunopeptidomics (28), have been covered in other review papers.

## Post-Processor Rescoring

The primary aim of DBSEs is to assign the correct peptide sequences to an experimentally acquired tandem mass spectrum (MS/MS). For this purpose, several scoring functions were developed that assess the similarity between experimental and theoretical MS/MS spectra. Thus, scoring functions are the bread and butter of proteomic research. One of the most well-known scoring functions used by several DBSEs, such as SEQUEST (33) and Comet (5), is cross-correlation (XCorr) (7), which generates the sum of all peaks that overlap between experimental and theoretical spectra under various displacements in mass-to-charge (m/z) dimension of the theoretical spectrum. Other DBSEs, such as Mascot (34) and MaxQuant (35), use a probabilistic scoring method that estimates the probability of observing a certain number of matching peaks by random chance.

Scoring peptide candidates in the form of theoretical spectra against experimentally acquired MS/MS spectra remains an active area of research. First, most scores developed in the past were not well calibrated within or across datasets and search spaces, which impairs their direct comparison. For instance, probabilistic scores are dependent on the length of the peptide, making it hard to compare scores of candidate peptides of different peptide lengths. Second, different scores provide complementary perspectives on the quality of a PSM. Consequently, most DBSEs today calculate multiple scores for each PSM to benefit from the different perspectives each score provides. As a result of both, scores need to be calibrated when attempting to perform a combined FDR estimation; otherwise, the scales, distributions, and directions of the various scores may hinder comparison within and between MS/MS analyses.

**Fig. 1:**
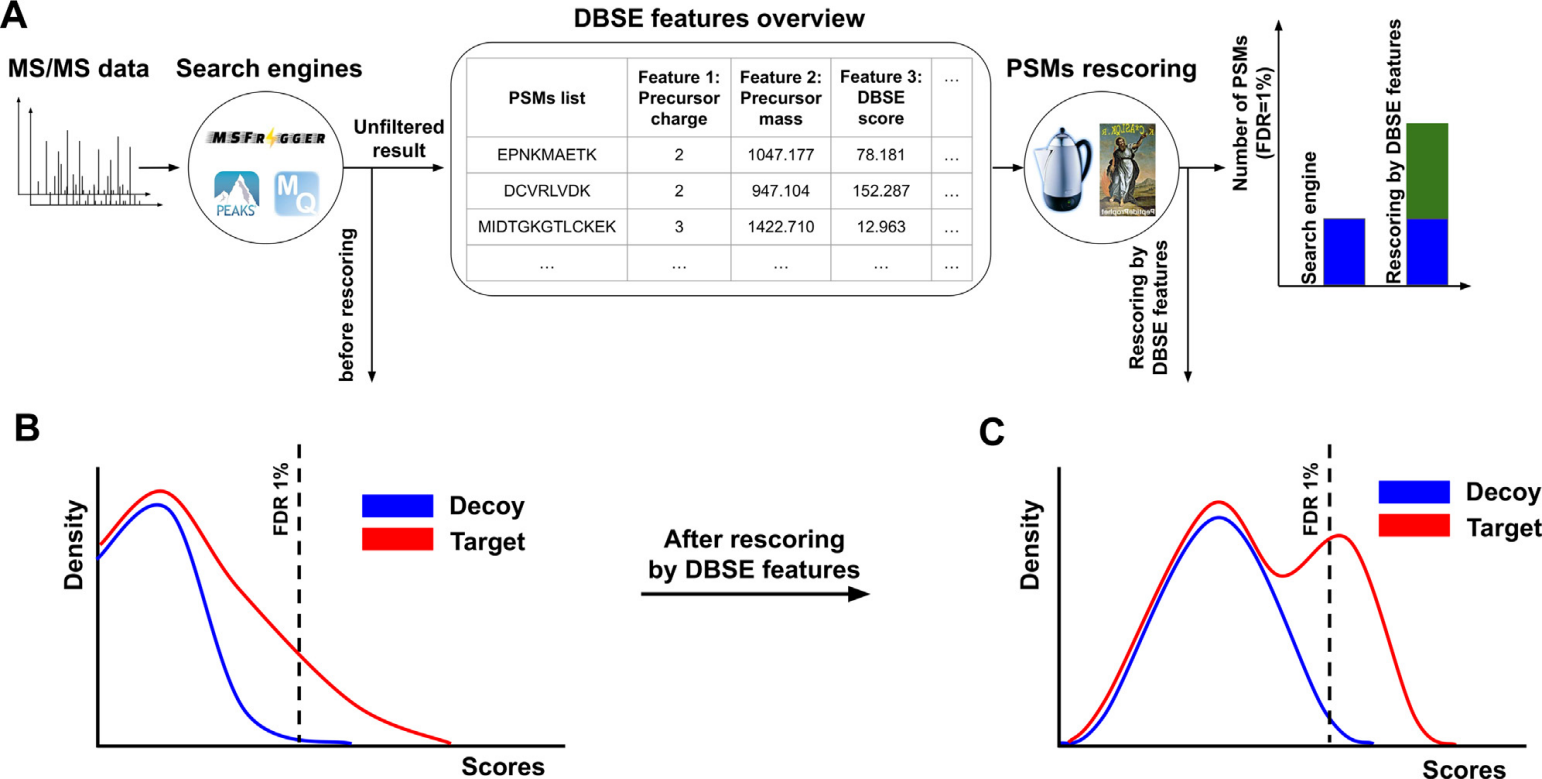
**Post-processor rescoring (PPR).***A*, tandem mass spectra (MS/MS) from data-dependent acquisition (DDA) data are most commonly processed using database search engines (DBSEs), such as MaxQuant, MSFragger, or PEAKS. The primary result of this process is a list of peptide spectrum matches (PSM), where each PSM has features attached, such as the precursor charge, the precursor mass, and the DBSE score. Today, most commonly this (unfiltered) list is handed over to post-processing rescoring (PPR) tools, such as PeptideProphet or Percolator, that train machine learning-based pipelines that classify PSMs into correct and incorrect PSMs including false discovery rate (FDR) estimation. *B*, an example of the DBSE score distribution of all PSMs before PPR. The dashed *black* vertical line indicates the cutoff required to achieve 1% PSM FDR. Note the poor separation between targets (*red*) and decoys (*blue*), indicating poor separation between correct and incorrect PSMs. *C*, example distribution of PSMs scores after PPR using all generated DBSE features. Note the increased separation between correct and incorrect PSMs step leads to better differentiation between target and decoy.

To overcome these constraints, rescoring, here referred to as post-processor rescoring (PPR), was introduced (Fig. 1*A*). PPR aims to calibrate and integrate multiple DBSE features (*e.g.*, DBSE scores, peptide length, etc) into a single feature (*e.g.*, score or probability) such that FDR estimation can be performed on all features combined. Machine learning is the method of choice for this task, as it allows the supervised or semi-supervised learning of dataset-dependent calibration and integration functions of these scores. Over the years, several PPR tools have been developed and integrated into multiple DBSEs as a post-processing step (Table 1). This has led to more standardized, comparable, and calibrated PSM scoring, enhancing the differentiation between correct (true positive targets) and incorrect (decoys) identifications compared to employing only a single DBSE score, and has significantly increased the number of confidently identified PSMs at a given FDR threshold in MS/MS analyses (Fig. 1, *B* and *C*).

**Table 1:** List of post-processor rescoring tools described in this review

Year of publication	Pipeline name	Employed classifier	Availability	License	Citations
2002	PeptideProphet (12)	Bayesian model	https://peptideprophet.sourceforge.net/	GPLv2	5498
2007	Percolator (11)	Support vector machine	https://github.com/percolator/percolator	Apache 2.0	2141
2011	Barista (48)	Feedforward neural network	https://bioinformaticshome.com/tools/proteomics/proteomics.html	Unspecified	39
2015	Nokoi (50)	L1-regularized logistic regression	Link unavailable	Apache 2.0	48
2019	Scavenger (52)	CatBoost	https://github.com/markmipt/scavager	Apache 2.0	48
2021	Mokapot (58)	Classifiers supported by scikit-learn	https://github.com/wfondrie/mokapot	Apache 2.0	25

### Workflow

The workflow of PPRs contains two main steps (Fig. 1*A*): (i) applying DBSE for identification and representation of PSMs by DBSE features, and (ii) PSM rescoring by ML models often in combination with FDR estimation. Briefly, experimental MS/MS spectra are initially analyzed *via* DBSE without applying an FDR filter, often even considering all PSM ranks for each MS/MS spectrum. This approach allows for an exploration of all possible PSMs resulting in a comprehensive list of potential PSMs, ensuring that all possible true (and false) positive identifications provided by a DBSE remain for the ML model. Unfiltered results (most commonly) must contain PSMs derived from the decoy database, which is essential for most ML models to learn to distinguish between correct and incorrect PSMs. Each PSM is represented by diverse DBSE features such as peptide characteristics (*e.g.*, length), precursor characteristics (*e.g.*, charge), matching characteristics (*e.g.*, mass error), and matching scores (*e.g.*, XCorr). In the second step, all labeled target and decoy PSMs with their corresponding DBSE features are submitted to the ML model for rescoring and classification (Fig. 1, *B* and *C*), leading to a higher rate of identified PSMs.

### Post-Processor Rescoring Tools

#### PeptideProphet

SEQUEST is one of the most well-known DBSEs used in proteomics and its influence on the field has substantially contributed to the maturation of proteomics (36). For match assessment between an experimental and theoretical spectrum, SEQUEST provides a set of scores, such as the previously mentioned XCorr and delta Cn, indicating how different the first PSM rank is from the second one based on their XCorr values. However, these scores were mostly considered separately for FDR estimation, which required applying different score cutoffs. PeptideProphet (12) was introduced as a PPR tool to jointly consider certain DBSE scores (*e.g.*, XCorr, delta Cn) for an efficient FDR estimation. This tool initially employs a discrimination function to combine DBSE scores into a single score. Each DBSE score is weighted, indicating its ability to discriminate between correct and incorrect PSMs. The weights are updated in an iterative process based on expectation maximization (EM) (37), containing two main steps. Firstly, the algorithm calculates the probability of each PSM being correct or incorrect using Bayesian theory based on the current discrimination function. Secondly, EM aims to update the weight of the discrimination function to better match its predictions to labeled PSMs, improving its ability to distinguish correct from incorrect PSMs. Through the years, PeptideProphet was integrated into various DBSEs such as SEQUEST, Mascot, and X!Tandem (38).

#### Percolator

Percolator (11), one of the most popular ML-based PPR tools available today, was developed to combine and integrate multiple scores provided by DBSEs. Rather than applying a statistical modeling approach, Percolator employs a support vector machine (SVM) to train a classifier to discriminate between correct and incorrect PSMs. Briefly, an SVM classifier attempts to locate a hyperplane that can optimally separate the classes while maximizing the distance between data points of different classes (39). Percolator was applied first on PSMs obtained from SEQUEST (40), which greatly enhanced the distinction between correct and incorrect matches. However, providing reliable generic training data is challenging, mainly because features that discriminate between correct and incorrect PSMs often vary between laboratories and experimental conditions. To address this, Percolator is trained on each dataset separately, using semi-supervised learning (11). For this, all PSMs from the decoy dataset are considered as the negative set, and high-scoring PSMs from the target dataset are used as the positive set. Cross-validation (41) is used to avoid overfitting and reliable PSM score estimation. Percolator (42) reports the q-values (FDR) and posterior error probabilities (PEP) for each identified PSM, peptide, and protein. To this day, Percolator remains one of the most widely used and trusted PPR in DBSEs and is used by, for example, MS-GF+ (43), Mascot, OMSSA (44), X!Tandem, Comet, and Tide (45).

#### Barista

Most commonly, researchers are eventually interested in the list of identified proteins. This is typically done in a two-step approach: peptides are identified using a DBSE and subsequently assembled into proteins by grouping identified peptides (46, 47). As a novel approach, Barista (48) considers protein identification as one problem. The core concept is to identify proteins directly from identified MS/MS spectra by employing a two-layer neural network for rescoring implementation at PSM, peptide, and protein levels. Briefly, each PSM is defined by a fixed set of DBSE features and subsequently scored. Since each peptide can be linked to multiple PSMs, the score of each peptide is specified as the highest score among its related PSMs. Ultimately, the protein score is calculated as the sum of connected peptide scores, indicating the probability of protein's existence in the sample. This Barista was integrated as a PPR tool into software named Crux, containing Comet and Tide as DBSEs.

#### Nokoi

All PPR tools discussed so far require target-decoy information for post-processing and FDR estimation. However, there are use cases, *e.g.*, proteogenomics (49), where the construction of a genome-sized decoy database is more problematic. To clarify, the decoy database should not contain any peptide found in the original genome while mimicking the nucleotide distribution of the target database. To address the aforementioned issues, a unique decoy-free approach (50) has been created in which each experimental spectrum is only compared to the target database. In this approach, PSMs are first ranked, and only top-ranking PSMs are considered correct matches, while the remaining PSMs are classified as incorrect. Next, all PSMs are represented by DBSE features and are rescored using a classifier called Nokoi (51), which works based on L1-regularized logistic regression techniques. Incorporating Nokoi into Mascot as DBSE has led to enhanced PSM identification.

#### Scavenger

Scavenger (52) introduces an alternative PPR tool designed to convert the set of scores provided by DBSE to a calibrated score. Regarding the applied target-decoy approach, Scavenger creates two separate groups where the first group includes all target PSMs with validation decoys, and the second group contains only training decoys. This approach prevents overfitting of the applied classifier, the open-source CatBoost gradient boosting library (53). After training, the classifier’s scores, representing the probability of a PSM belonging to the decoy group, are employed to calculate the posterior error probabilities of PSMs. Scavenger supports multiple DBSEs such as X!Tandem, Comet, MSFragger (54), Morpheus (55), IdentiPy (56), and MS-GF+.

#### Mokapot

To improve the peptide identification rate in some specific areas such as protein-RNA interaction (57) and single-cell proteomics, the adaptable Python version of Percolator, called Mokapot (58), was proposed. One of the specific features of Mokapot is that users can take advantage of non-linear classifiers for the PPR process. To examine the capability of Mokapot, both SVM and the non-linear classifier called XGBoost (59) were employed to identify modified peptides derived from protein-RNA crosslinking. The results indicate that applying XGBoost results in more accurate detection of RNA-cross-linked peptides compared to the SVM alone. A further use-case highlighted was single-cell proteomics, which can be challenging when only a limited number of PSMs are provided *via* DBSEs for PPR. To address this, Mokapot offers an approach in which the identified PSMs from all experiments are merged into a unified dataset to train a joint model. Then, the trained joint model can be applied to each experiment separately to improve the peptide identification rate.

## Data-Driven Rescoring Pipelines

Despite the advances in proteome coverage by integrating PPR into DBSEs, most DBSEs have considered only the presence of peaks in MS/MS spectra and, if at all, only partly incorporate fragment ion intensity information into their PSM scoring. Over the years, some developments aimed to integrate more intensity information of MS/MS spectra. For example, C. Narasimhan *et al*. (60) introduced a new technique that converts peaks in a spectrum (their intensities, density, and peak positions) into a probability profile to calculate scores. In another work, D. Tabb *et al*. (61) first categorize peaks in a spectrum into different classes based on their intensity. Then, a statistical approach is applied for scoring so that matched peaks from higher-intensity classes contribute more than those from lower-intensity classes to the final score.

Although these approaches showed a decent gain in performance on certain samples, DBSEs often struggle when dealing with very large search spaces. Briefly, the large search space reduces the specificity of DBSEs since there are highly similar peptide sequences that could differ only in a few low-intense peaks, which can increase the chance of false positive identification. For example, in immunopeptidomics, the MHC-bound peptides of interest do not follow any known proteases, which not only changes the MS/MS spectra pattern but also significantly increases the search space that has to be considered by DBSEs. A similar scenario happens within metaproteomics data analysis, where the primary purpose is to investigate proteins from multiple organisms rather than studying proteins of one species, requiring a comprehensive protein database that must be considered in the search space. In addition to the search space issue, DBSEs have also experienced difficulty in analyzing spectra from low-abundant samples in certain domains, such as single-cell proteomics and clinical-based datasets (62). This difficulty arises because low-input samples contain a large number of low-abundance proteins and thus the resulting peptides give rise to lower-quality MS/MS spectra. As a result, spectra often contain fewer detectable peaks in comparison to spectra acquired from bulk samples, decreasing the sensitivity of DBSEs.

One effective solution for dealing with the above-mentioned complex datasets is to employ machine learning models designed for the prediction of peptide properties, such as MS/MS intensity (19, 63) and RT (19, 21), to calculate and add certain data-driven features into PPR tools. In one of the pioneering studies (64) aimed at enhancing protein identification in mycobacterial strains, the MS2PIP (65) model was employed to predict the b- and y-ion fragment intensities of peptide candidates extracted from search results provided by Mascot. Then, multiple data-driven features were generated by comparing experimental and predicted peak intensities of MS/MS spectra and passed to the Percolator for PPR. After this initial exploration, subsequent studies built on this approach and achieved impressive results (66, 67). Following this, various data-driven rescoring (DDR) pipelines (23, 24, 25, 26) have been developed to bridge the gap between DBSEs, machine learning models, and PPR, aiming to make the process more user-friendly and efficient for broader adoption (Table 2).

**Table 2:** List of existing data-driven-(like) rescoring pipelines designed for boosting proteome coverage

Year of publication	Pipeline name	MS/MS prediction	RT prediction	Additional predictions	PPR tool	Fully supported PTMs	Augmented PTM support	Supported DBSEs	Availability	Primary evaluation data
2018	CharmeRT (68)	-	Elutator’s RT (68)	-	Elutator (68)	Unknown	any (by transfer learning)	MS Amanda	https://ms.imp.ac.at/?goto=charmert	tryptic peptides
2019	MS-Rescue (101)	-	-	-	-	any	-	PEAKS, MaxQuant	http://www.cbs.dtu.dk/cgi-bin/sw_request?msrescue	immunopeptidomics
2019	MHCquant (105)	-	-	MHCflurry (BA) (106)	Percolator (11)	any (by ignoring PTMs)	-	Comet	https://github.com/ewels/nf-core-mhcquant/tree/master	immunopeptidomics
2019	DART-ID (29)	-	(Alignment model (29))	-	Custom bayesian model (29)	any	any	MaxQuant	https://github.com/SlavovLab/DART-ID	single cell proteomics
2020	DeepRescore (71)	pDeep2 (64)	AutoRT (74)	-	Percolator (11)	carbamidomethylation, oxidation, TMT, iTRAQ, phosphorylation	-	MS-GF+, Comet, X!Tandem, MaxQuant	https://github.com/bzhanglab/DeepRescore	immunopeptidomics
2021	INFERYS rescoring (27)	INFERYS	INFERYS	-	Percolator (11)	carbamidomethylation, oxidation	-	Sequest HT as part of Proteome Discoverer	Closed source/Available in Proteome Discoverer	immunopeptidomics
2022	MS2Rescore (23)	MS2PIP (83)	DeepLC (21)	-	Percolator (11), Mokapot (58)	carbamidomethylation, oxidation, TMT, iTRAQ, phosphorylation	any (by shifting and extrapolation)	MaxQuant, PEAKS, PeptideShaker, Mascot, MS-GF+, X! Tandem, Sage, Customizable	https://github.com/compomics/ms2rescore	proteogenomics, immunopeptidomics, metaproteomics, single cell proteomics
2022	AlphaPeptDeep (25)	AlphaPeptDeep (25)	AlphaPeptDeep (25)	AlphaPeptDeep (CCS/IM)(25)	Percolator (11)	carbamidomethylation, oxidation, N-term acetylation, phosphorylation, ubiquitination, dimethyl	any (by transfer learning)	AlphaPept, MaxQuant	https://github.com/MannLabs/alphapeptdeep	various proteases, immunopeptidomics, timsTOF PASEF
2022	InSPIRE (88)	Prosit (19), MS2PIP (83)	Prosit (19), Pyteomics (89)	netMHCpan (BA) (22)	Percolator (11), Mokapot (58)	carbamidomethylation, oxidation, modifications supported by MS2PIP	Unknown	MaxQuan, PEAKS, Mascot	https://github.com/QuantSysBio/inSPIRE	immunopeptidomics
2022	DeepSCP (92)	SampleRT (92)	DeepSpec (92)	-	LgbBayes (92)	carbamidomethylation, oxidation, N-terminal acetylation	Unknown	MaxQuant	https://github.com/XuejiangGuo/DeepSCP	single cell proteomics
2023	Oktoberfest (24)	Prosit (19), MS2PIP (83), AlphaPeptDeep (25)	Prosit (19), DeepLC (21), AlphaPeptDeep (25)	AlphaPeptDeep (CCS/IM)(25)	Percolator (11), Mokapot (58)	carbamidomethylation, oxidation, TMT, iTRAQ, TMTpro	deamidation (by shifting)	MaxQuan, MSAmanda, Mascot, MSFragger, Sage, Customizable	https://github.com/wilhelm-lab/oktoberfest	various proteases, proteogenomics, immunopeptidomics, metaproteomics, timsTOF
2023	MSBooster (26)	DIA-NN (69)	DIA-NN (69)	DIA-NN (CSS/IM) (69)	Percolator (11)	carbamidomethylation, ubiquitination, oxidation, phosphorylation, N-terminal acetylation	any (by shifting)	MSFragger	https://github.com/Nesvilab/MSBooster	immunopeptidomics, single-cell proteomics, timsTOF PASEF

Prediction tools listed in parenthesis are not considered property predictors but are used in a similar context. The list of fully supported PTMs denotes the modifications for which the property prediction models were trained, while the modifications listed in augmented PTM support are generated by, *e.g.*, shifting the expected fragment masses by the delta mass of the modification, transfer learning models with user data or the ability of models to extrapolate to unknown PTMs. A dash denotes no support for PTMs. Note that if all (denoted as any) PTMs are supported then additional support by augmentation is not necessary.

### Workflow

**Fig. 2:**
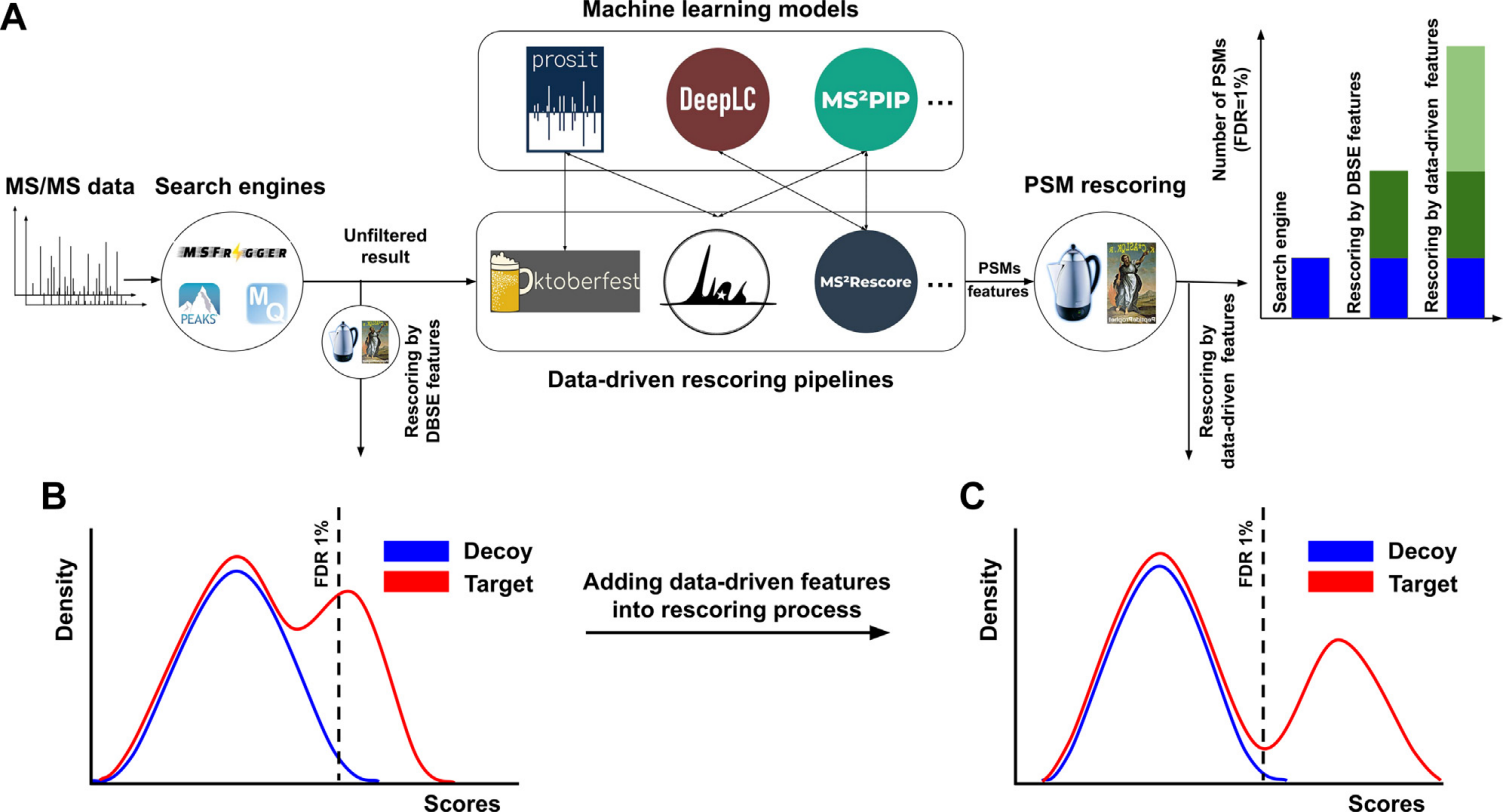
**Data-driven rescoring (DDR) approach**. *A*, in data-driven rescoring (DDR), such as Oktoberfest, InSPIRE, and MS2Rescore, unfiltered DBSE results are further processed by additional features such as a similarity metric between predicted (*e.g.* by Prosit, DeepLC, and MS2PIP) and observed spectra or retention time of PSMs. PPR is applied on the resulting list of features to classify and estimate the FDR of the provided PSMs. In contrast to filtering correct matches solely based on the DBSE score (*blue bar* in barchart) and PPR applied on DBSE features (*blue* + *dark green*), DDR (*blue* + *dark green* + *light green*) typically achieves the highest number of identified PSMs. *B*, example distribution of PSM scores after PPR using all generated DBSE features. The dashed *black* vertical line indicates the cutoff required to achieve 1% PSM FDR estimated by the target (*red*) decoy (*blue*) approach. *C*, example distribution of PSMs scores after DDR. Note the further increased separation power between correct and incorrect PSMs and the increase in confidently identified targets.

At its core, DDR pipelines follow a similar workflow consisting of three main steps (Fig. 2*A*). First, experimental MS/MS spectra are examined by DBSEs without applying filters (explained in PPR section) ensuring the investigation of all possible PSMs. Second, for each PSM provided, additional matching properties are generated as features (or scores) that compare experimental peptide properties to predictions. Currently, DDR workflows rely on four main property predictions: (i) MS/MS, (ii) RT, (iii) collisional cross section (CCS) or ion mobility (IM) (68), and (iv) binding affinity (BA) (22) predictions. For MS/MS, RT, and IM predictions, scores can be derived that assess the level of agreement (or disagreement) between the prediction and observed values, for example, the absolute difference between predicted and observed retention time. Property predictions such as binding affinity, used in immunopeptidomics, are often used as independent features as peptides that are not predicted to be presented by the human leukocyte antigen (HLA) complex may have a higher chance of being false positives. Similar concepts could be applied to *e.g.*, digestibility or detectability predictions (69), that indicate how likely it is that a certain peptide (and thus PSM) is observable by LC-MS/MS. Lastly, PSMs with their corresponding data-driven features are submitted to PPR, mostly Percolator, to classify correct and incorrect PSMs. The newly calculated matching scores can either be concatenated with or entirely replace the DBSE scores. Multiple studies have shown the benefits of DDR compared to only utilizing DBSE features (Fig. 2B-C), resulting in the identification of a higher number of PSMs, peptides, and proteins at a desired FDR and even allowing more stringent filtering while maintaining a high number of true positives (19, 23).

### Prominent Data-Driven Rescoring Pipelines

#### DeepRescore

DeepRescore (70) was developed to improve the sensitivity and reliability of MHC-bound peptides and neoantigen identification. This pipeline requires MS/MS data in MGF format and PSM identification results from supported DBSEs, including MS-GF+, Comet, X!Tandem, and MaxQuant, as input. DeepRescore calculates two data-driven features named delta RT and normalized spectral angle (SA) (71) in conjunction with pDeep2 (MS/MS predictor) (72) and AutoRT (RT predictor) (73). Briefly, delta RT and SA show the difference between predicted and observed RT and the similarity between experimental and predicted MS/MS, respectively. It supports MS data derived from labeling techniques, particularly Tandem Mass Tag (TMT) and Isobaric Tags for Relative and Absolute Quantification (iTRAQ), and also supports Methionine oxidation as variable modification. In addition to the data-driven features, DeepRescore considers DBSE features (*e.g.*, DBSE scores and peptide charge) for PSM representation. Ultimately, all PSMs with their corresponding features (11–15 features depending on the utilized DBSE) are submitted to Percolator for PPR. Recently, DeepRescore was further extended to improve the identification rate in phosphoproteomics (74). Concerning benchmarking, DeepRescore was tested on two public immunopeptidomics datasets (75, 76). Using DeepRescore increased the number of unique peptides by up to 109% at 1% FDR compared to DBSEs, such as MS-GF+, Comet, X!Tandem, and MaxQuant.

#### INFERYS Rescoring

INFERYS rescoring (27) is integrated into Proteome Discoverer and designed for PSM identification refinement of Sequest HT results (77) by employing an internal deep learning model, which was inspired by Prosit and developed for MS/MS and RT prediction. INFERYS requires the peptide sequence, precursor charge, and collision energy (CE) as input to generate predictions. To maximize the fragment intensity prediction performance, INFERYS first calibrates the CE used for prediction for each MS file. Briefly, the pipeline selects top-ranking identified PSMs provided by Sequest HT and then employs INFERYS to predict fragment intensities in various CEs. The CE that leads to the highest SA is selected as the input for the model for subsequent data-driven rescoring. Next, INFERYS calculates multiple data-driven features per PSM, such as SA, and merges them with the DBSE features. In terms of modification, this pipeline covers carbamidomethylation of Cysteine and Methionine oxidation. Finally, Percolator is applied for PPR and FDR estimation. INFERYS was evaluated on a dataset containing HLA peptides (78) increasing the number of identified peptides at 1% FDR by 50%.

#### MS2Rescore

MS2Rescore (23) represents a modular package that expects DBSE results, as well as either MGF files, mzML files, or raw files as input for the DDR. It supports multiple search engines, including MaxQuant, PEAKS (79), PeptideShaker (80), Mascot, MS-GF+, X!Tandem, and Sage (81). For DDR, MS2Rescore generates multiple features assessing the similarity between observed and predicted MS/MS spectra (*e.g.*, Pearson correlation and cosine similarity) utilizing MS2PIP (82), whereas the features are calculated only for b-ions, y-ions, or both. Furthermore, MS2Rescore calculates RT-dependent features, *e.g.*, delta RT, utilizing DeepLC (21). In the latest version of MS2Rescore (83), a CCS/IM predictor termed ionmob (84) was integrated that is used to generate new features scoring the difference between the expected and the observed CCS/IM of a peptide. The resulting set of data-driven features is then combined with the DBSE features (*e.g.*, precursor mass, number of missed cleavages) and is transferred to Percolator or Mokapot for PPR. For validation, datasets containing HLA class I and class II peptides (85) were re-analyzed by (i) MS2Rescore and (ii) MaxQuant with Percolator. The results showed that MS2Rescore (i) improved the identification rate of PSMs by 46% compared to the second method (ii).

#### AlphaPeptDeep

AlphaPeptDeep (25) requires MS raw data and identified PSMs provided DBSEs such as MaxQuant and AlphaPept (86). AlphaPeptDeep is shipped with multiple pre-trained models for MS/MS, RT, and CCS/IM prediction. It natively supports a variety of post-translational modifications (PTMs) including carbamidomethylation, oxidation, N-term acetylation, ubiquitination, dimethyl and phosphorylation. Furthermore, this pipeline is also able to handle other PTMs by applying transfer learning. Briefly, AlphaPeptDeep selects a certain number of PSMs with and without modifications provided by the DBSE to fine-tune the pre-trained models, which enhances the prediction performance of the existing models and extends the supported PTMs. Next, AlphaPeptDeep calculates 61 data-driven features, including correlations of predicted and experimental MS/MS spectra, delta RT, and delta CCS. As the final step, Percolator is used for PPR and FDR estimation. For the validation, two public datasets (75, 85) were analyzed in various ways: (i) by MaxQuant as DBSE, (ii) MaxQuant with Prosit and Percolator, and (iii) AlphaPeptDeep. The results showed that AlphaPeptDeep boosted the number of identified peptides by 100% and 7%, respectively.

#### InSPIRE

InSPIRE (87) is optimized especially for boosting peptide coverage in immunopeptidomics. It supports MS data in mzML and MGF format and supports various DBSEs, including PEAKS, MaxQuant, and Mascot. InSPIRE offers a more flexible and user-friendly pipeline than others by allowing the user to select either Prosit or MS2PIP for MS/MS prediction, and Prosit or Pyteomics (88) for RT prediction. Furthermore, NetMHCpan (22) is integrated into InSPIRE to predict BA between HLA peptides and the HLA-complex. In terms of supported modifications, this pipeline is restricted to carbamidomethylation and oxidation when using Prosit, while it can cover more modifications (*e.g.*, TMT, iTRAQ, and iTRAQphospho) by MS2PIP. InSPIRE generates multiple data-driven features, such as Spearman correlation and SA between predicted and experimental spectra, predicted BA, and delta RT. Furthermore, InSPIRE calculates a unique additional feature called Prosit-delta. Briefly, the authors argued that some peptide sequences should be considered less reliable when minor sequence alterations result in only minor changes in the SA, compared to peptide sequences where *e.g.* amino acid substitutions highly affect the SA. To avoid predicting MS/MS spectra of all possible sequence variations, the Prosit-delta predictor was developed that aims to predict the expected difference in SA. Finally, all data-driven features are combined with the DBSE features (approximately 41 features in total). InSPIRE was evaluated on an immunopeptidomics dataset (89) that was analyzed by various pipelines: (i) Rescoring using the Prosit web server (90), (ii) InSPIRE-MS2PIP, (iii) InSPIRE-Prosit, and (iv) InSPIRE-Prosit-affinity. Applying (ii), (iii), and (iv) led to around 3%, 4%, and 8% increase in PSMs at 1% FDR compared to (i), respectively.

#### DeepSCP

DeepSCP (91) has been specifically developed to increase proteome coverage in single-cell proteomics (SCP). It reads identified PSMs from MaxQuant results and then applies SampleRT and DeepSpec, developed along DeepSCP, for data-driven feature generation. Briefly, SampleRT calculates statistical features of peptides’ experimental RTs across various SCP measurements in order to predict RTs by ElasticNet (92), a linear regression model. DeepSpec is a deep learning model designed and integrated into DeepSCP for MS/MS spectra prediction. These models can handle carbamidomethylation, oxidation, and N-terminal acetylation as PTMs. Similar to other DDR pipelines, delta RT and cosine similarity are used as one of the main features for PSM representation. Ultimately, DBSE and data-driven features are merged (24 features in total) and used as input for PPR performed by LgbBayes, developed alongside DeepSCP. LgbBayes utilizes the LightGBM (93) algorithm, a gradient-boosting framework built on decision trees. To evaluate DeepSCP’s performance, multiple datasets (94, 95) were analyzed in different ways: (i) MaxQuant as DBSE, (ii) Mokapot as PPR, (iii) and DeepSCP. In (94), DeepSCP exhibited a 30% increase in PSMs over MaxQuant and a 20% increase over Mokapot at 1% FDR. Similarly, for (95), DeepSCP showed a 19% and 15% increase compared to MaxQuant and Mokapot in PSMs at 1% FDR, respectively.

#### Oktoberfest

Oktoberfest (24) covers multiple DBSEs, including Mascot, MSFragger, MSAmanda, and MaxQuant. This pipeline applies the deep learning model Prosit that predicts RT and MS/MS spectra, accessible through an online prediction service called Koina (https://github.com/wilhelm-lab/koina). To achieve optimal performance of Prosit, similar to INFERYS rescoring, Oktoberfest initially performs CE calibration for each MS data file and then generates data-driven features in conjunction with Prosit. Concerning RT, Prosit predicts the indexed retention times of peptides which are then aligned by Oktoberfest to the experimental RT based on a spline regression method. Furthermore, Oktoberfest calculates several features related to MS/MS prediction, such as SA, the number of observed fragment ions with non-zero predicted intensity, and the number of observed fragment ions without predicted intensity. Similar to MS2Rescore, this is done for only b-ion, only y-ions, and both. In total, Oktoberfest generates 92 features per PSM. For PPR and FDR estimation, Percolator or Mokapot can be used and supports carbamidomethylation, oxidation, TMT, iTRAQ, and TMTpro. Prior studies have shown Oktoberfest’s capability to increase the number of identified PSMs across diverse data types. For example, a dataset containing HLA-I immunopeptides (85) was analyzed first by MaxQuant+Percolator and second by Oktoberfest. The results revealed a 296% increase in identified PSM at 1% FDR with Oktoberfest, compared to using only MaxQuant and Percolator.

#### MSBooster

FragPipe is a suite of computational tools enabling comprehensive analysis of mass spectrometry-based proteomics data, covering multiple applications such as PSM identification by MSFragger and Percolator for PPR. To benefit from DDR, MSBooster (26) was developed and placed between MSFragger and Percolator that allowing the efficient analysis of challenging data such as immunopeptidomics, single-cell proteomics, and data acquired on, for example, the timsTOF platform. MSBooster utilizes DIA-NN (68) to predict MS/MS spectra, RT, and CCS/IM. DIA-NN supports diverse modifications, including oxidation, phosphorylation, carbamidomethylation, ubiquitination, and N-terminal acetylation. For MS/MS spectra similarity, MSBooster calculates the unweighted spectral entropy (96). Regarding RT, the experimental RT is initially calibrated against the predicted RT using a regression model, leading to the calculation of delta RT. The same process is also done for IM. MSBooster was evaluated on various datasets, such as HLA-I immunopeptides (85), in two different ways: by MSFragger with Percolator, and then by MSBooster. The results demonstrated a 183% increase at 1% FDR in the number of identified peptides by MSBooster compared to the application of MSFragger and Percolator.

### Data-Driven-Like Rescoring Pipelines

In addition to DDR, a number of noteworthy additional rescoring pipelines were published that do not follow the definition of DDR pipelines, but heavily rely on or utilize ML and AI models to improve the number of confidently identified peptides.

#### CharmeRT

Chimeric spectra represent complex MS/MS spectra containing more than one peptide. This problem arises from the fact that multiple peptides with similar RT and m/z can be co-isolated during LC-MS/MS (97). CharmeRT (67) was designed to identify multiple peptides per spectrum and was integrated into the DBSE MS Amanda (8). To address the issue of chimeric spectra, MS Amanda initially performs a first peptide search and then removes all peaks related to the confidently identified peptides from the corresponding spectra. Subsequently, a second peptide search is performed, which aims to identify the secondary component of potentially chimeric spectra. For this, CharmeRT utilizes Elutator’s RT model inherited from SSRCalc (98). This model considers peptide length, the isoelectric charge, and other features as input and predicts their hydrophobicity index. This index is then linearly mapped to retention time, leading to the calculation of delta RT as the primary data-driven feature. Eventually, CharmeRT applies Elutator, a PPR similar to Percolator. To benchmark CharmeRT’s capability, a dataset containing a tryptic digest of HeLa cell lines (99) was analyzed in different ways: (i) Mascot, (ii) MaxQuant, (iii) MS Amanda, (iv) MS Amanda with Elutator as PPR (but without the RT model), and (v) CharmeRT (MS Amanda with Elutator with the RT model). The results highlighted that CharmeRT achieved the highest number of identified PSMs at 1% FDR (∼38k PSMs) compared to the other approaches, which identified (i) ∼18k PSMs, (ii) ∼17k PSMs, (iii) ∼19k PSMs, and (iv) ∼35k PSMs.

#### MS-Rescue

MS-Rescue (100) is a pipeline optimized for the improvement of immunopeptidomics analysis. This pipeline follows a unique approach compared to other pipelines where only low-confidence PSMs (above a 1% FDR) are targeted for rescoring. Briefly, MS-Rescue requires a list of peptides with their corresponding DBSE scores, such as the Andromeda score used by MaxQuant. High-confidence peptides are used as the training set, while low-confident peptides are considered the recovery set in this workflow, a set of peptides that can be rescued. The peptides from the training set are submitted to GibbsCluster (101) to perform binding motif clustering. Peptides that cannot be grouped with others are removed. After that, the grouped peptides are tagged with their corresponding clusters and used to train NNAlign (102). The trained NNAlign model is then used to score the recovery set. The peptides from the recovery set that achieve the highest scores are included in the final list of identified peptides. To evaluate the pipeline, three immunopeptidomics datasets from various species, including human (100), mouse (100), and cattle (103), were analyzed by MS-Rescue, leading to a 32%, 27%, and 14% increase in the number of identified PSMs at FDR 1%, respectively, compared to applying only DBSE.

#### MHCquant

MHCquant (104) is designed for the identification and quantification of HLA peptides, which requires a protein database in FASTA format and MS data as input. This pipeline initially employs Comet as DBSE to provide unfiltered PSMs and then combines DBSE features with predicted BA derived from MHCflurry (105). Finally, all PSMs with their features are submitted into Percolator for rescoring implementation. For benchmarking, a dataset containing nine peripheral blood mononuclear cells and four JY cell lines (human lymphoblastoid B-cell line) was analyzed by MHCquant and compared to various DBSEs, including MS-GF+, PEAKS, SequestHT, and Mascot. The results indicated that MHCquant outperformed the other DBSEs and improved the rate of identified peptides (around 7% to 90% depending on the used DBSE), which were predicted as HLA-binders through various HLA binding predictors, such as NetMHCpan, NetMHC (22), SYFPEITHI (106), MHCflurry, and PickPocket (107).

#### DART-ID

DART-ID (29) was introduced as a pipeline specifically designed for the refinement of PSM identification of MS data coming from single-cell proteomics by mass spectrometry (SCoPE-MS) (108). Here, similar samples are analyzed on identical LC-MS/MS systems, resulting in multiple RT estimates per peptide including technical variation among all experiments. DART-ID initially performs a global RT alignment to compensate for systematic RT variation, leading to high-accuracy RT estimation. Ultimately, DART-ID rescores each PSM by integrating RT information using a custom bayesian model (12). To demonstrate the performance of the package, DART-ID was tested on data derived from U-937 and HEK-293 cell lines (109), where the authors could show an increase in identified PSMs at 1% FDR by ∼50% compared to applying MaxQuant alone. In addition to SCoPE-MS data, both label-free (110) and TMT-labeled MS data (111) were analyzed by DART-ID, resulting in a 30% to 50% improvement in identified PSMs at 1% FDR.

## Future Perspective

### Data-Driven Rescoring vs. Predicted Spectral Library Searching

The integration of fragment intensity (and retention time) information into the matching process was a key argument in favor of spectral library searching. Unlike DBSEs, which heavily rely on the presence of numerous fragments to generate high scores for PSMs, SLSEs primarily score the intensity pattern of fragments (*e.g.*, *via* cosine spectrum similarity). Consequently, SLSEs were known to achieve higher sensitivity and typically outperform DBSEs, especially when dealing with low-quality spectra resulting from (1) low-abundance proteins (2), samples with high dynamic range (3), ion suppression, or (4) poorly fragmenting peptides. Therefore, it is not surprising that DDR has demonstrated advantages over classic DBSEs in similar applications. Simultaneously, the integration of peptide property predictions resulted in substantially improving the ability to distinguish correct from incorrect matches in large search spaces, enhancing the specificity of peptide identifications below a given FDR threshold (23, 112). Particularly in cases where samples of low input amount and large search spaces are investigated, as is the case in immunopeptidomics, DDR can outperform DBSEs by almost an order of magnitude (28).

While both challenges can, in principle, be addressed by SLSEs, the results were previously dependent on peptides being present in the used spectral library; otherwise, confident identification could not be achieved. With the increasing availability of high-quality predictors, partially driven by DDR pipelines in recent years, complete spectral libraries for any use case can now be generated at the press of a button (32). This has sparked the development of novel SLSEs (113, 114), enabling the efficient utilization of proteome-scale libraries and incorporating advances in fragment indexing strategies to remain competitive in terms of computational resources required.

With DDR becoming readily available to any user, facilitated by the growing number of available pipelines and their versatile applicability, the commonly used arguments in favor of SLSEs or DBSEs are becoming less one-sided, as the disadvantages of the two approaches are diminishing if not completely abolished. While the majority of users currently rely on DBSEs, particularly in the context of DDA data analysis, SLSEs could emerge as the new workhorse for proteomics researchers.

An argument in favor of SLSEs utilizing proteome-scale predicted libraries is their ability to correctly score theoretical spectra against experimentally acquired spectra *a priori* by considering only the expected non-zero intensity fragments and not all possible (including zero intensity predicted) fragments. This will decrease the score of otherwise incorrectly ranked peptide candidates which are not expected to generate certain fragments. Particularly in DDR, scores that assess the number of observed but zero-intensity predicted fragments, have been shown to be beneficial in classifying incorrect PSMs (19). This contrasts with the most commonly used scores in DBSE that often sum up the number of expected fragments, designed to promote PSMs to correct identifications. Integrating such features in DBSEs will require on-the-fly predictions, often limited by the availability of dedicated hardware as high-quality or high-performance predictors often require graphics processing units (GPUs).

While SLSEs inherently benefit from comprehensive predicted libraries with reference-like quality (115), one main challenge still remains: The generated libraries may become prohibitively large, affecting storage, sharing, and utilization. In a recent study, a predicted spectral library was used to showcase the performance of Mistle in metaproteomics (114). The generated library contained ∼28M spectra and required ∼50 GB of storage. While the authors demonstrated that Mistle works well in this use case, extrapolating such a library to all possible HLA class I peptides from a canonical human proteome would require generating, storing, and handling an msp file with more than 250 GB in size, storing upwards of 150 M spectra for ∼45 M unmodified peptide sequences. This size would increase even further when considering possible PTMs (116), mutations (115), or even cryptic HLA peptides (32). Furthermore, such libraries will likely have to be generated for every mass spectrometer and various instrument settings separately in order to maintain the high specificity of the library. The latter, in particular, may substantially hinder the use of SLSEs since even slight differences in data acquisition can result in large differences in the data. Previous observations have noted that the effective CE applied for fragmentation (despite being nominally set the same) varies across instruments, even of the same type, and within the same instrument over time (117). As a result, data acquired over a longer period of time may require multiple instances of a library generated at various CEs. Additionally, it has been reported that spectra acquired from bulk samples may show different fragmentation characteristics than spectra acquired from single-cell data (118), which would further increase the complexity and size of predicted libraries. The advantage of DDR is that predictions are retrieved in real-time and thus could be produced in a dataset-, raw file- and even spectrum-specific context; increasing runtime and hardware requirements.

DDR can be applied on more than the first-ranked PSM (multi-rank DDR) generated by a DBSE. However, the specifics of the implementation of such an approach matter, and more research is necessary to investigate its potential benefits and drawbacks, since this may result in a too-optimistic FDR estimation. The three most important questions to be addressed in multi-rank DDR are: (1) are multiple PSMs per spectrum allowed to pass the FDR cutoff, (2) is this process precursor aware, and (3) can fragments (or their intensity) be used for more than one PSM. If, for example, only one PSM - the highest scoring after rescoring - is picked, likely little negative effect on the quality of the FDR estimation is expected. This process essentially results in a re-ranking of PSMs and allows DDR pipelines to pick better (*e.g.*, estimated by spectral similarity) PSM for a spectrum than the first-ranked PSM proposed by the DBSE (19). This may inherently be the case for SLSE using predicted libraries as those would already rank PSMs based on spectral agreement, rather than a heuristic score. Allowing multiple PSMs per spectrum but only one for each detected precursor is similar to so-called second peptide searches (6, 67) and addresses the presence of chimeric spectra, spectra that contain fragments from multiple precursors (119). It was suggested to assess the FDR for first peptides (typically peptides matched to the precursor which was meant to be fragmented by the mass spectrometer) and second peptides (typically a co-eluting precursor within the isolation window) separately to maintain good estimation quality. This can be done with DDR but can also be implemented in SLSEs similar to current best practices in DBSEs. However, it is paramount in all cases to monitor if peaks or portions of the intensity of peaks are used for multiple PSMs that survive FDR filtering. To illustrate this with an extreme case, if the first and second peptides explain the same precursor and share all (but very few) fragments. In such a case, one should refrain from calling both peptides. For either DBSEs or SLSEs, high-quality predictions may allow the accurate estimation of how much-shared intensity of peaks is possible and allowed.

At present, both approaches, DBSEs using DDR and SLSEs using predicted libraries, have an equal chance of becoming the new workhorse in proteomics research. DDR, as the logical continuation of DBSEs, does not come with the same overhead as SLSEs (*e.g.*, in the form of storage space consumption) at the cost of specificity during initial scoring. Certainly, both approaches rely on, or at least very strongly benefit from (1), readily available (2), high quality (3), high performance, and (4) highly versatile peptide property predictors. With those being readily available today, ease of use and the resilience of a tool to new data may be the key to success.

### Limitations and Opportunities of Data-Driven Rescoring

Comparing the performance of DDR pipelines becomes increasingly challenging as users are left with a plethora of choices. The choice of a DDR pipeline explicitly dictates the supported search engines and available prediction models. Furthermore, it implicitly dictates the types and assumptions of features calculated by the pipeline. This renders fair comparison difficult as, in good experimental practice, the various differences in DDR pipelines cannot be investigated in isolation in order to assess their importance and relevance in DDR. While some tools (*e.g.*, MS2Rescore) would allow a more systematic comparison of DDR on different search engines and others (*e.g.*, INSPIRE) would allow the comparison of different prediction models, a systematic comparison of all effects is not easily possible given the current landscape of tools.

An opportunity of DDR is its ability to facilitate the aggregation, combination, and selection of the best PSMs from multiple DBSEs, a task which has been shown to increase the number of identifications, but remains challenging due to the different features and scores being generated by multiple search engines (80). DDR pipelines could act as a unifier, providing DBSE-independent scores that allow PPR to select and combine the results and, thus, excel from the different benefits DBSEs provide.

Further, DDR could also offer a potential for improving peptide identification from data-independent acquisition (DIA) data where fragmentation is repeatedly performed on all peptide ions from a wider isolation window resulting in intentionally chimeric spectra, unlike the selective approach done in data-dependent acquisition (DDA). Among available DDR pipelines, only MSBooster currently offers rescoring of peptide identifications from DIA data. To clarify, MSBooster-based rescoring was demonstrated on spectrum-centric results originating from either MSFragger-DIA (120), which directly identifies peptides from DIA data, or DIA-Umpire (121), which generates pseudo-MS/MS spectra from DIA data. This restriction may largely be the result of two limitations when aiming to apply rescoring on DIA data. First, the spectrum-centric nature of DDR may require substantial changes to the workflow since most tools for DIA data perform a peptide-centric analysis (122) today. In addition, such tools often do not export unfiltered search results including decoy matches, required for DDR, and the use of sample-specific libraries (subsets of expected true positive target peptides) generated as part of the processing workflow may further limit the application of DDR since a limited number of false positive targets is present - and sometimes even replaced by the shuffled version of the expected true positives. Second, rescoring DIA-NN results with MSBooster must be performed with great caution, since MSBooster utilizes DIA-NN predictions. This is the case for any DDR pipeline run after a peptide-centric analysis which used the same predictor to generate the library. Without further research, it may also be advisable to not even use a different predictor that was trained on the same data as this may cause data leakage. Extending other DDR pipelines to offer rescoring of DIA data would enable further research, build additional confidence in DIA data analysis, and allow orthogonal validation of results.

While DDR pipelines are becoming readily available to users, requiring little to no prior experience in bioinformatics (*e.g.*, *via* user interfaces in the case of MS2Rescore), two remaining limiting factors may still be (1) the dependency on GPUs for peptide property prediction, and (2) the computational performance requirements of pipelines. Both are somewhat connected as running larger DL models is typically orders of magnitude slower when done on CPUs in comparison to GPUs, while fast CPU-based classic ML-based models often exhibit poorer accuracies. While not all applications of DDR may require optimal prediction performance, further research is necessary to investigate which use cases require which level of prediction certainty to carefully balance processing speed (and hardware requirements) against the comprehensiveness (and correctness) of the results. However, for DDR to fully mature, crucial processing steps, mostly peptide and protein quantification, are missing in all pipelines.

The boost in identifications as a result of the increased sensitivity of DDR pipelines also provides new opportunities to re-assess if currently used data acquisition protocols and MS settings are optimal. Particularly the superior performance on low abundant peptide species could result in decreasing fill time requirements for medium or higher abundant peptide species, further increasing the rate at which MS/MS events can be triggered. A further area of research could be to explore to which extent the increase in specificity by incorporating fragment intensities during the matching process could decrease the importance of high mass accuracy, ultimately leading to faster (more frequent) MS/MS events, which may further increase sensitivity for low input samples (123).

Another boost in the sensitivity and specificity of DDR could be achieved by integrating more informative scores and features. For MS2Rescore, the observed gains in DDR performance over alternatives were postulated to be driven by the additional features it calculates, rather than a superior performance of MS2PIP (23). Since all DDR pipelines calculate rather straightforward and interpretable features, *e.g.*, the correlation of intensity values between predicted and observed spectra (including or excluding certain types of ions), recent advances in generating novel matching scores could result in even higher gains. Over the last years, various such methods have been released that may provide even better granularity in separating correct from incorrect matches (96, 124, 125), particularly in cases where only low intense fragments differentiate between many possible peptide candidates. As previously observed for HLA class I peptides (90), and most commonly an issue when performing site localization of PTMs (126), slightly different peptides may result in almost identical spectra, resulting in essentially identical correlation measures. Without scores to discriminate amongst such cases, PPR will struggle to pick the correct identification and the results may be driven by random chance as a result of measurement uncertainties. ML-based scores could be designed to focus on small differences, rather than being driven by the high abundant peaks in a spectrum, and could even be fine-tuned to specific use cases (127).

### Novel Predictors to Further Boost Performance

The most prominent and predictive features include MS/MS peptide fragment intensities and peptide RT, while some tools also incorporate properties such as CCS/IM. Apart from these well-established features, further properties could be investigated for incorporation into the DDR process.

One of the most prominent properties readily available is the observed precursor charge state of measured peptides. Accurately predicting the primary charge state or charge state distribution of a peptide of interest can reduce the number of potential candidates to investigate by a factor of 1 to 5, having implications not only for runtime but also overall sensitivity/specificity due to a decrease in search space. Properties with similar effects on the number of candidates to be considered are, for example, digestibility (69), detectability (69) and ionization efficiency (69, 128). The boundaries between these terms are not well defined and may largely depend on the source of data used for training. For example, a model prediction peptide detectability may or may not include digestibility (whether a peptide is generated during digestion) or ionization efficiency (how well peptides can be ionized) depending on whether it was trained on quantified synthetic peptides or digested proteins. However, the combination of all such predictors and respective features could result in utilizing the proteotypicity (129) of a peptide, here referring to the chance of observing a peptide precursor under the assumption that its protein is present in the sample. This could offer the ability to penalize candidates of low probability or prioritize peptides expected to be present with high probability. While such property predictors were developed in the past, an integration into DDR has not yet been implemented. An opportunity may arise here by integrating approaches such developed in Barista or Percolator to perform protein inference while peptide identification is done and thus could effectively use peptide detectability predictions.

Recent advances in protein function, secondary and tertiary structure prediction from *e.g.*, protein language models (130), could spark the development of novel proteotypicity predictors. Stretches of proteins predicted to be inaccessible to standard workflows (131) are likely useful pieces of information relevant for peptide detectability prediction (132). Further, protein structure and function predictions may be particularly relevant in the context of generating, scoring, and analyzing peptides with PTMs (133, 134), as only some modifications are possible at some residues within a protein, depending on the localization of the protein.

Some of the properties mentioned here (*e.g.*, precursor charge, digestibility, flyability, etc) could also be used to efficiently filter predicted spectral libraries. To the best of our knowledge, all predicted libraries generated for SLSEs were built with the heuristic that every peptide can be present in any charge and modification state, irrespective of its composition (*e.g.*, two missed cleavages) and localization (*e.g.*, within the transmembrane domain) within a protein. This could very effectively reduce the generated libraries to more manageable sizes. However, such predictors must be used with caution as incorrectly excluding peptide candidates, *e.g.*, based on the assumption that certain PTMs may not be possible on certain peptides, may critically affect our ability to discover unknown or unexpected biology.

A particular challenge when aiming to accurately predict peptide properties such as precursor charge or flyability remains experimental variability. While models for MS/MS prediction can be trained to account for instrument variability - implemented in some DDR pipelines by performing, for example, CE calibration - similar approaches are still lacking for other peptide properties. For retention time, most often DDR pipelines perform RT alignments, mapping predicted (indexed) RTs to experimentally acquired RTs. However, most models predict RT under “common” settings as certain aspects of RT variability cannot be solved by “simply” performing (non-linear) alignments (135). Peptide properties governed by processes upstream of LC-MS/MS analysis are less well understood and, for instance, the particular kinetics governing digestion efficiency or peptide solubility are much harder to predict accurately. This is in large part driven by the lack of high-quality annotated training data, as datasets do not contain detailed descriptions of the precise protocols experimentalists rarely annotate the wanted (or unwanted) modifications applied. There is an urgent need by the machine learning community to get access to well-annotated data for model training (136).

To enable DDR pipelines to benefit from other accurate peptide property predictions that currently cannot be calibrated as easily as, *e.g.*, CE, fine-tuning (also referred to as refinement learning or transfer learning) is a viable approach. Briefly, fine-tuning refers to adjusting a pre-trained model on custom data to improve its accuracy to the specific protocol used. This and similar concepts have been successfully used in proteomics to achieve high-quality predictions in the absence of large amounts of training data (72, 137). A future direction to integrate accurate predictions of less well-studied properties could, thus, be to integrate real-time fine-tuning into DDR pipelines, where a subset of high-confidence PSMs are used as training data to fine-tune generic pre-trained models. This would further enable users to generate lab-, protocol-, or project-specific models and thus also predicted spectral libraries, further circumventing the need to generate complete libraries containing spectra of unlikely observable precursors.

### New Frontiers in ML-Guided Feature Generation

Apart from the development of novel peptide property predictors for DDR pipelines, a new frontier in DDR pipelines is the need to make the model’s limitations more transparent. DDR pipelines facilitate the development of new machine- and deep-learning models, yet, the underlying learned mechanisms of the trained models remain mostly unclear. Users rely on such “black boxes” and develop trust in the quality and correctness of their predictions. However, in which circumstances such models fail to generate high-quality predictions is under-researched since publications showcasing novel predictors focus primarily on the many cases where models perform well. Most models exhibit inferior performance for some subset of the data, which is particularly visible when spectral similarity is investigated. While it is easy to argue that such cases may simply be the result of incorrect assignments in the training data - all training data used today is generated by DBSEs, which generate some portion of incorrect assignments even when synthetic peptides are analyzed (19) - it may also hint at implicit biases of models where certain (unknown) subclasses of peptides cannot be predicted well.

The opposite may also be the case, where models perform exceptionally well on a subset of the peptides. In the worst case, this is the result of overfitting, when a model begins to memorize the training data by heart rather than learning to generalize to unknown cases. This can happen when models contain more parameters (weights) than required, a common problem in machine learning (138). Here, model sizes are continuously increased in an attempt to reach higher accuracy, without comparing against baseline models to assess whether a simpler model with fewer parameters may perform equally well (136, 139). A prominent example may be IM, which is highly correlated to the precursor's mass and charge, allowing a simple machine learning model to perform very comparably to complex deep learning models (140).

Model overfitting can be avoided by separating data into a training set, a validation set for evaluation during training, and a holdout (or test) set to assess the predictions of the model on unseen data, primarily to test its generalization capabilities toward novel data and avoid a model specifically optimized for a certain validation set. Still, it is difficult to guarantee the independence of the holdout set, for example, due to inadvertent contaminants (139). This can lead to a false estimation of a model’s generalization capabilities (141).

To compensate against potential biases of models with respect to overfitting and lack of generalization, developers of peptide property predictions should make all training, validation, and testing data publicly available, particularly the lists of precursors used (139). This is not only to enable fair and unbiased model performance evaluations between two or many models - which should ideally be done only on peptides contained (if at all) exclusively in the test set - but also to enable DDR pipelines and particularly users of DDR pipelines to do expectation management on the quality of the predictions.

DDR pipelines may benefit from knowing whether a precursor rescored in a dataset was used for training the peptide property predictor since this might influence the score distributions of PSMs. Different thresholds and expectations may need to be applied, for example, a higher threshold for precursors part of the training data, as prediction quality for precursors not in the training data may be lower (19). Similar biases in score distributions may be the result of class imbalance in the training data, since a model may have not seen as many MS/MS spectra for long peptides as it did for shorter peptides. Similar to what was observed for DBSE’s matching scores (*e.g.*, probabilistic matching scores), the performance of peptide property predictions is often dependent on the length and charge state of peptides, resulting in the deteriorating performance of the predictors. While such effects are known and, thus, can be mitigated by PPR, other potentially more subtle biases (*e.g.*, in the frequency of amino acids) may not be captured by PPR as uncertainties in the confidence of the predictions are neither estimated nor provided. A particular challenge relevant for, for example, immunopeptidomics is the use of sample-specific predictors, such as binding affinity (28). Depending on the level of overfitting of such models, DDR may largely only recover known binders with an impaired ability to identify unknown binders. Potentially even more detrimental is overfitting to the true target peptides. Similar problems may, however, also arise when utilizing peptide detectability predictors trained on *e.g.* different sample preparation workflows. Any sample-specific predictor bears the risk of biasing the analysis, which is not expected to be the case for predictors focusing on less sample-specific properties, *e.g.* MS/MS intensity.

Overfitting peptides from the target database holds the biggest risk when applying machine learning since PPR relies on the fact that the features provided do not encode target or decoy information explicitly. If this is the case, overfitting of the PPR model (*e.g.*, Percolator) can occur, which can result in too optimistic FDR estimations. In the worst case, a perfect separation between targets and decoys could be achieved and not, as aimed for, a separation between correct (true positives from the targets) and incorrect (decoys and false positives from the targets) matches. A commonly raised concern is the unknown performance and potential biases of peptide property prediction models on decoy peptides, as such models have typically only seen peptides from a target database during training. A model may have inadvertently learned the distribution and frequency of amino acids in a target database and may not be able to generalize to decoy peptides. While, to the best of our knowledge, this has not been shown so far, further research is required to rule out the possibility of information leakage (target and decoy peptides) to PPR. A possible route forward to circumvent such issues may be double-decoy approaches as implemented in Scavenger, or to avoid the problem altogether by using decoy-free FDR estimation approaches, for example, implemented in Nokoi.

The above challenges should all be considered in future developments of DDR pipelines. Not only should users be aware of common pitfalls, but also developers should make an effort to increase the transparency and reliability of DDR. For this, scientists in the field of machine learning (*e.g.*, for property prediction), software development (*e.g.*, for DDR pipelines), biochemistry (*e.g.*, for peptide flyability), and mass spectrometry (*e.g.*, MS/MS data) must work together to fully understand the causes, the estimation, and the steps to circumvent the interaction of aleatoric and epistemic uncertainty. Uncertainty prediction is an active area of research and has been shown to increase the interpretability of results achieved in downstream applications using machine learning (142). Besides uncertainty prediction, DDR pipelines should adopt methods of out-of-distribution detection (143), the process of statistically or adversarially detecting samples that are far away from the training distribution. For example, a model trained exclusively on tryptic peptides may not perform well or even result in incorrect predictions on non-tryptic peptides and should be used with great caution for DDR in such cases. Out-of-distribution detection is critical to ensure the reliability (and safety) of machine learning systems. Combining this with uncertainty prediction could be a powerful mechanism to safeguard users from unknowingly selecting an inappropriate model while at the same time guiding users toward the model with the least amount of uncertainty on their particular use cases.

## Conclusion

Machine learning-based post-processing of data has paved the way for major improvements in our ability to analyze proteomics data sparked almost 2 decades ago. Today, the generation of high-quality matching features using deep learning-based peptide property predictors facilitated by PPR holds the potential to lead to similar improvements. In fact, current research shows that across all majority applications of LC-MS/MS in protein research, DDR is able to increase the number of confidently identified peptides and, thus, inferred proteins. While the degree of increase varies, typically showing the largest increases for large search spaces or lower quality spectra due to low input amounts, the possibility to further reduce false positives while keeping the majority of true positives is beneficial for any workflow. Likely, the next generation of search engines will include similar or even more advanced processes for scoring peptides (144). However, while it remains open whether DBSE or SLSE will become new workhorses in proteomics, the boundaries between the two will certainly become more blurry and the advantages shown by DDR may result in a complete fusion of the two approaches.

While collecting the information relevant for DDR pipelines, two issues became apparent. First, comprehensive and easy-to-access documentation of usage is often lacking, limiting users in their ability to pick and adopt these new approaches. The lack of documentation was particularly apparent when collecting information about the supported PTMs, which is often not directly available. Without a strong IT background, users may struggle to pick and apply the appropriate tool for their use case. Tools developed in academic environments often do not follow established standards for software development, documentation of code, and best practices for communication between developers and users. Having open-source code and issue trackers, as well as documentation on code-sharing platforms such as GitHub, helps mitigate these problems since users can publicly ask questions and share issues with each other and with the developers. As a result, this allows the community to contribute and steer developers to new features as they are desired/required by the users. A good indication for selecting the right tool can therefore also be how active the developers are in maintaining the software and communicating with the users, as active participation of the users is equally important for future developments. Second, most DDR pipelines lack fully integrated quantification pipelines which impairs their direct adoption in the majority of proteomics use cases where quantification is paramount. The usefulness of most DDR pipelines was shown in immunopeptidomics, where quantification of peptides is often not used or even required. Noteworthy exceptions are the closed source pipeline FragPipe, where the DDR tool MSBooster can be applied to MSFragger search results using DIA-NNs predictions, and MHCquant. Despite peptide identification, concepts from data-driven rescoring have been successfully applied to other areas, such as improving and correcting modification site localization (74, 127), improving the accuracy of *de novo* peptide sequencing (145, 146), and increasing the number of confidently identified cross-linked peptides (147). It is thus very probable that DDR will become the new standard in MS-based proteomics.

## Data Availability

All data are located in the manuscript and supporting information.

## Conflict of interest

The authors declare the following financial interests/personal relationships which may be considered as potential competing interests: M. W. is a co-founder and shareholder of MSAID GmbH and OmicScouts GmbH, with no operational role in both companies.
